# Insect-induced tree mortality of boreal forests in eastern Canada under a changing climate

**DOI:** 10.1002/ece3.988

**Published:** 2014-05-16

**Authors:** Xiongqing Zhang, Yuancai Lei, Zhihai Ma, Dan Kneeshaw, Changhui Peng

**Affiliations:** 1 Department of Biological Sciences, Center for Forest Research, University of Quebec at MontrealC.P. 8888, Succ. Centre-Ville, Montreal, H3C 3P8, Canada; 2 Research Institute of Forestry, Chinese Academy of ForestryBeijing, 100091, China; 3 Research Institute of Forest Resource Information Techniques, Chinese Academy of ForestryBeijing, 100091, China

**Keywords:** Carbon dynamics, climate change, eastern Canada's boreal forest, forest insect, spruce budworm, tree mortality

## Abstract

Forest insects are major disturbances that induce tree mortality in eastern coniferous (or fir-spruce) forests in eastern North America. The spruce budworm (SBW) (*Choristoneura fumiferana* [Clemens]) is the most devastating insect causing tree mortality. However, the relative importance of insect-caused mortality versus tree mortality caused by other agents and how this relationship will change with climate change is not known. Based on permanent sample plots across eastern Canada, we combined a logistic model with a negative model to estimate tree mortality. The results showed that tree mortality increased mainly due to forest insects. The mean difference in annual tree mortality between plots disturbed by insects and those without insect disturbance was 0.0680 per year (*P* < 0.0001, *T*-test), and the carbon sink loss was about 2.87t C ha^−1^ year^−1^ larger than in natural forests. We also found that annual tree mortality increased significantly with the annual climate moisture index (CMI) and decreased significantly with annual minimum temperature (*T*_min_), annual mean temperature (*T*_mean_) and the number of degree days below 0°C (DD0), which was inconsistent with previous studies (Adams et al. 2009; van Mantgem et al. 2009; Allen et al. 2010). Furthermore, the results for the trends in the magnitude of forest insect outbreaks were consistent with those of climate factors for annual tree mortality. Our results demonstrate that forest insects are the dominant cause of the tree mortality in eastern Canada but that tree mortality induced by insect outbreaks will decrease in eastern Canada under warming climate.

## Introduction

Forest insects play important roles in terrestrial ecosystems. There are many reports of outbreaks of insects and their devastating impact on forests (Logan et al. 2003; Wermelinger 2004). In the Canadian boreal biome, forest insects (particularly defoliators) are a major disturbance agent that affect productivity through reduced growth, increased tree mortality, and complicated interactions with other disturbances (Candau and Fleming 2011). As an estimation, the average annual volume of wood lost due to forest insects in Canada during the period 1982–1987 was about 1.4 times the losses from forest fire and 0.22 times the annual loss to logging (Hall and Moody 1994). Forest insect outbreaks have an adverse effect on the balance of carbon sequestered by forests (Volney and Fleming 2000). Landscape-scale tree mortality from insect herbivores also releases carbon to the atmosphere, which can exacerbate climate change (Kurz et al. 2008).

Tree defoliation is the major insect disturbance of boreal forests in eastern Canada, especially due to the spruce budworm (SBW), which is one of the most destructive native insects in the northern spruce and fir forests of eastern North America (Kneeshaw et al. 2011). The possibility of increasing tree mortality due to insect outbreaks in boreal forests is a particular concern because boreal forests are recognized as an important “tipping element” of the Earth's climate system (Lenton et al. 2008). Recent studies indicate that threats to the forest carbon sink as a result of tree mortality caused by forest insects in North America have unexpectedly increased in the past decade. Kurz et al. (2008) found that the cumulative impact of the mountain pine beetle outbreak for the period 2000–2020 will be 270 megatonnes (Mt) of carbon as the forest will be converted from a small net carbon sink to a large net carbon source both during and immediately after the outbreak. Dymond et al. (2010) reported that the ecosystem carbon stock during modeled SBW outbreaks was reduced on average by 2 Tg C year^−1^ for the entire simulated area.

It is also possible to investigate climate influences across large geographic areas. Some authors have suggested that insect outbreaks will increase in frequency and severity as climate changes (Fleming et al. 2002). Range shifts are also predicted with outbreaks occurring beyond traditional limits as climate becomes more hospitable (Régnière et al. 2012). These range shifts can expose secondary hosts to outbreak herbivory which may be exacerbated by the outbreaking insects displacing more quickly than controlling parasitoids (Stireman et al. 2005). Tree hosts are also affected by climate and may be more vulnerable to insect attack if it stressed by non-optimal climatic conditions (Mattson and Haack 1987).

Although there are a number of studies on tree mortality induced by SBW and other insects, these are mostly restricted to one tree species, one insect species, or one political jurisdiction (province or state) (Batzer 1973; MacLean and Piene 1995; Simard and Payette 2001; Bouchard and Pothier 2010). However, to our knowledge, no comparable studies of chronic long-term changes in mortality rates induced by forest insects have been conducted in natural (unmanaged) boreal forests using long-term forest permanent sampling plots (PSPs). The objective of the study was to investigate the relationship between forest insects and tree mortality, as well as to consider the effect of other variables, such as stand characteristics, location variables, and climate variables. We also evaluated the effects of climate variables on forest insects in order to understand the potential impact of climate change on insect-caused tree mortality. The work in the study was logistically conducted in four steps: Firstly, we modeled tree mortality as a function of insect disturbance; secondly, we modeled tree mortality as a function of climatic variables; thirdly, we modeled insect disturbance as a function of climatic variables; lastly, we modeled the relationship of insect disturbance with climate to tree mortality by other variables (province, dominant tree species, diameter class, stand age, elevation, and latitude).

## Data and Methods

### Forest permanent sample plot data

Forest permanent sample plots (PSPs) from eastern Canada's boreal forest regions were strictly selected based on the following criteria: (1) All plots were in natural forest stands, which we defined as stands that developed naturally rather than after forest management, such as thinning, harvesting, or other silvicultural treatments. (2) To avoid changes in tree mortality caused by other disturbance, only plots with no evidence of fire, flooding, storms, or disturbances other than caused by insects were chosen. (3) All plots were measured at least three times. (4) Complete tree mortality records were required in the study. In addition, the tree diameter measurements in all plots were conducted after tree height reached 1.3 m during the first census. (5) Individual trees must have been clearly tagged and repeatedly measured. (6) To reduce random variation in plot-level demographic data, only plots with a large number of live trees (≥50, which is based on our calculation of averaged tree number over minimum sample plots [plot size = 0.04 ha]) at their initial census were used. (7) To obtain climatic data for each plot, the spatial location of all plots was required. These selection criteria are critical as the plots managed by provincial forest inventories and permanent sample plots were originally established for diverse purposes, such as to investigate forest growth and yield, describe different stages of forest development, document the dynamics of certain forest types, or explore forest dynamics along environmental gradients.

**Figure 1:**
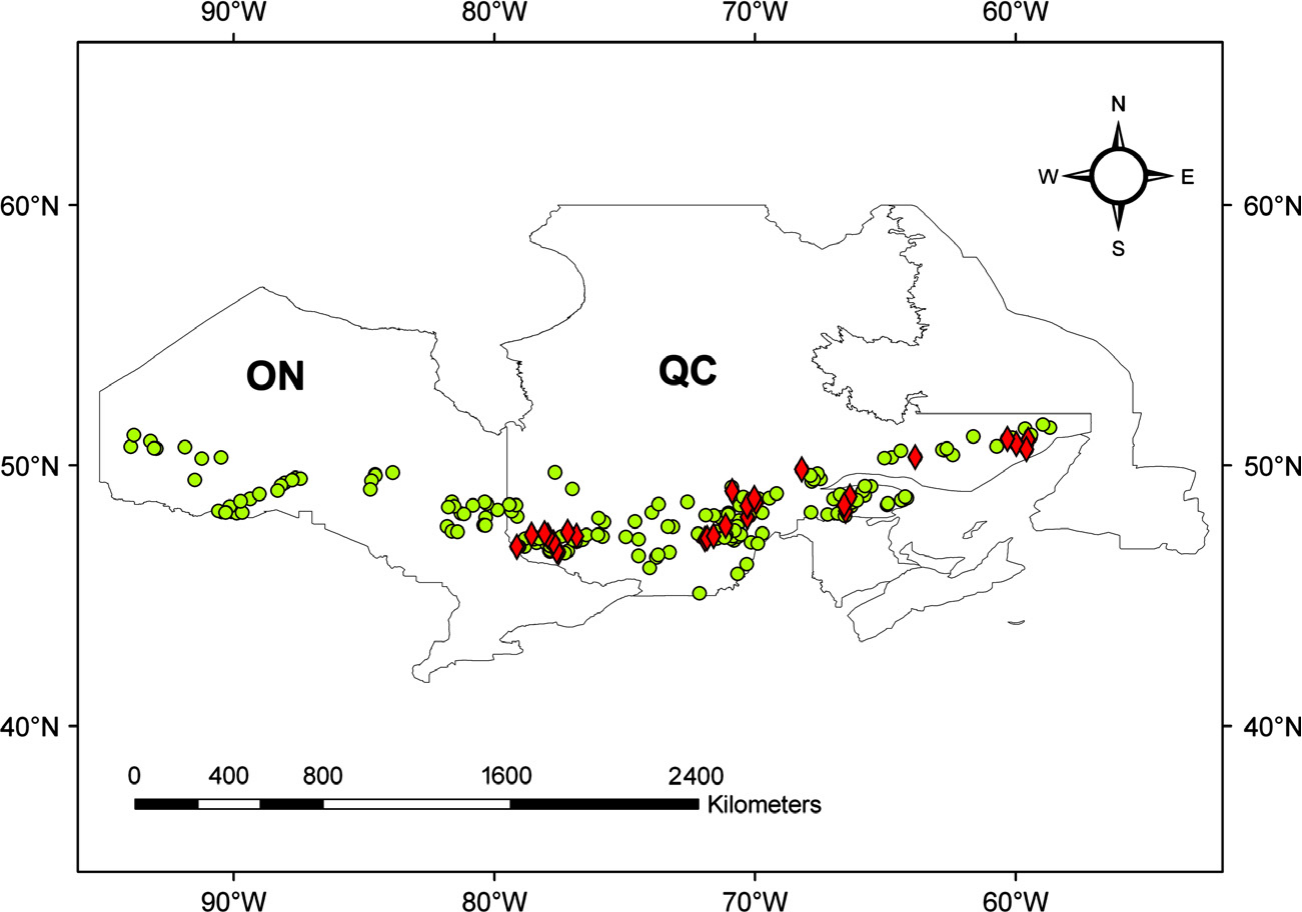
Location of plots disturbed by forest insects in Ontario (ON) and Quebec (QC). Plots with >75% trees disturbed by insects are in red, and <75% trees are in green.

For this study, we selected and thoroughly reviewed data from PSPs in Ontario (Hayden 1995) and Quebec (Duchesne and Ouimet 2008). We did not use PSPs data from New Brunswick because most plots in this province are not considered to be part of the boreal forest type. Although there were many boreal PSPs in each province (more than 4000 plots for Ontario and about 12,000 plots for Quebec), most of the plots did not meet our criteria. In the end, 1701 plots met our criteria, of which 226 plots are disturbed by forest insects. Table S1 summarizes the key characteristics of the 226 plots, and their locations are shown in Fig. 1. In this dataset, the forest was mostly disturbed by the SBW, which is consistent with Fleming (2000) who noted that the SBW is the most important insect disturbance in Canada's boreal forest, especially for eastern Canada, and that mortality caused by other insects is trivial. For our analyses, forest insect outbreaks were treated as a dummy variable (0: undisturbed by forest insect; 1: disturbed by forest insect).

### Climate data and climatic variables

To obtain the climatic variables associated with the individual plots, the daily 10-km raster gridded climate dataset for Canada from 1961 to 2003 (Daily 10 km Gridded Climate Dataset: 1961–2003^2007^) was used, which contains data with daily maximum temperature (°C; *T*_max_), minimum temperature (°C; *T*_min_), and precipitation (mm; *PRE*) for the Canadian landmass south of 60°N. The 10 × 10 km grids were interpolated from daily Environment Canada climate station observations using a thin-plate-smoothing spline-surface-fitting method implemented by the ANUSPLIN V4.3 software (Hutchinson 2004). The annual climate moisture index (CMI) (Hogg 1997) was used to indicate the annual climatic water deficit. Positive CMI values indicate relatively moist conditions, and negative CMI values indicate relatively dry conditions. The annual mean temperature (°C; *T*_mean_) and annual precipitation were also calculated. To explore the effect of low temperature and growing season on annual tree mortality and forest insects, the number of degree days below 0°C (DD0) was calculated. To model changes in tree mortality and magnitude of forest insect outbreaks as a function of climatic variables, the mean values of the annual climatic variables across all years within each census interval for a given plot were taken.

### Statistical models

#### Relationship between tree mortality and forest insect disturbance, climatic variables and other variables

We used the same statistical models of van Mantgem et al. (2009) and Peng et al. (2011), which were simple, appropriate to the data, and capable of detecting mortality rates. To estimate changes in mortality rates, we modeled the rate as a logistic function exp(*β*_0_ + *β*_1_*x*_*i*_+*γ*_*i*_)/(1 + exp(*β*_0_ + *β*_1_*x*_*i*_ + *γ*_*i*_)), where *i* represents plot number, *x* represents the independent variables, such as disturbance or not by forest insects, climate variables, stand characteristics, and location variables for plot *i*, *β*_0_ and *β*_1_ are regression parameters, and *γ*_*i*_ is a random effect parameter among the multiple plots. We applied a statistical model to our data where *n*_*i*_ was the number of trees alive for the *i*th plot, and *m*_*i*_ represents the corresponding mortality rate:

*m*_*i*_|*γ*_*i*_ ∼ negative binomial with mean *n*_*i*_*p*_*i*_ and variance


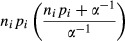
(1)


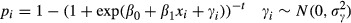
(2)

where *p*_*i*_ represents the probability of mortality over the census interval *t*. The random intercept parameter *γ*_*i*_ follows a normal distribution. The negative binomial distribution is an extension of the Poisson distribution with *α* > 0 representing overdispersion (Liu and Cela 2008).

#### Relationship between forest insect disturbance and climatic variables and other variables

Two levels of forest insect disturbance are defined based on the percentage of disturbed trees by forest insects: 0 (less than 75% of the trees in a plot are disturbed by forest insects); 1: (more than 75% of the trees in a plot are disturbed by the forest insect) (Blais 1981; Bergeron and Leduc 1998). Because forest insect disturbance data in this study are a binary variable, we modeled annual forest insect magnitude with the logistic model.


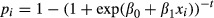
(3)

where *p*_*i*_ represents the annual forest insect magnitude over the census interval. Because we found there was a random effect of multiple plots on forest insect disturbance, we used a nonlinear logistic model to analyze the effect of forest insect disturbances.

We used maximum likelihood to estimate model parameters producing the most likely tree mortality and forest insect disturbance, when compounded based on the length of the census intervals, and which best corresponded to the rates observed in the data. We also used a *T*-test to compare the effects of dominant species and diameter classes on tree mortality and forest insect disturbance.

#### Estimation of carbon loss to insects

We used aboveground stand biomass measured in ton carbon ha^−1^ as a proxy because it can be converted to a measure of carbon stored in tree biomass (Fahey et al. 2010). Although aboveground biomass is just one of several carbon pools in forest ecosystems, it can represent a significant proportion of forest carbon. We calculated the carbon stock through aboveground biomass multiplying by a carbon conversion factor of 0.5. Based on stand arithmetic mean diameter of each plot, we first calculated the average plot-level biomass by summing the dry biomass components of its wood, bark, foliage, and branches according to published Canadian national equations (Lambert et al. 2005). We then calculated the average carbon stock per ha.

## Results and Discussions

### Modeling tree mortality as a function of insect disturbance

Based on equation 1, we found that tree mortality rates increased with the occurrence of forest insect disturbances (Table 1). In the Canadian boreal biome, forest insects (particularly insect-caused defoliation) constitute a major disturbance that increases tree mortality (Candau and Fleming 2011). Sustained defoliation can result in tree deformations, reduced growth, top kill, and tree mortality. The mean difference in annual tree mortality between plots disturbed by insects and not disturbed by insects was 0.0680 (*T*-test, *P* < 0.0001), and the carbon stock loss was about 2.87t C ha^−1^ year^−1^ larger than in natural forests.

**Table 1:** Fixed effects in the generalized nonlinear mixed models describing annual tree mortality as a function of forest insect disturbance (0: noinsect; 1: insect) and *N* is the number of plots

Variable	*β*	*P*	*N*
Insect disturbed or not	1.3861	<0.0001	1701

### Modeling tree mortality as a function of climatic variables

Annual tree mortality increased with Annual CMI and decreased with mean annual *T*_min_, annual *T*_mean_ and DD0 (Table 2), which was inconsistent with previous studies (Adams et al. 2009; van Mantgem et al. 2009; Allen et al. 2010). This could be explained by the fact that unlike western Canada, tree mortality in the eastern Canadian boreal forest was not caused by climate warming and drought. In these forests, disturbance by forest insects was the main factor resulting in tree mortality.

**Table 2:** Annual tree mortality and forest insect disturbance by climate variables. Annual CMI: annual climate moisture index, Annual mean *T*_min_, annual mean minimum temperature; Annual *T*_mean_, annual mean temperature; DD*0*: degree days below 0°C

	Tree mortality		Forest insect disturbance	
		
Variables	*β*	*P*	*β*	*P*
Annual CMI	0.0071	0.0175	0.0293	0.0010
Annual mean *T*_min_	−0.2293	<0.0001	−0.4891	0.0045
Annual *T*_mean_	−0.2022	0.0014	−0.4665	0.0066
DD0	−0.0012	0.0136	−0.0028	0.0003

### Modeling insect disturbance as a function of climatic variables

Forest insect disturbance was positively related to annual CMI and negatively to annual mean *T*_min_, annual *T*_mean_, and DD0 (Table 2). This could explain the contradiction of our results with those from other studies (van Mantgem et al. 2009; Allen et al. 2010). In addition, there was a positive relationship between the trends in both tree mortality and forest insect disturbance and climate variables (Figs. 3).

**Figure 2:**
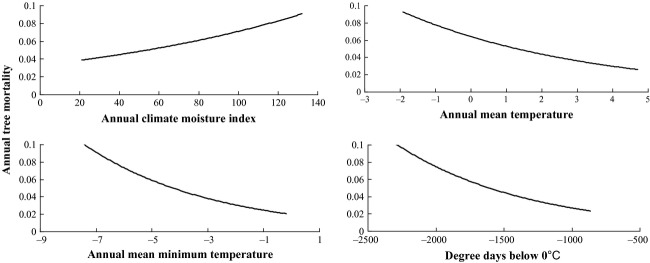
Tree mortality trends with climate variables.

**Figure 3:**
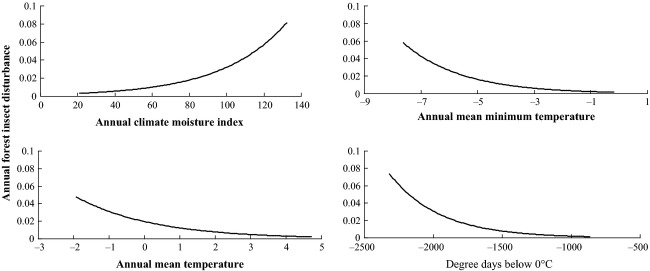
Forest insect disturbance trends with climate variables.

Drought creates stress in trees and lowers their defenses, making them less resistant to defoliating insect attacks (the plant-stress hypothesis) (Mattson and Haack 1987; Croisé and Lieutier 1993; Rouault et al. 2006), as well as to attacks by bark-beetles (Caldeira et al. 2002). Early studies of the SBW also suggested an association between the beginning of an outbreak and a series of preceding dry summers (Ives 1974; Lucuik 1984). In contrast, in this study, tree mortality increased with high humidity (high CMI). Both Royama (1984) and Martinat (1987), however, questioned the relationship between SBW outbreaks and drought noting flaws in the methods of the earlier studies. Morris and Fulton (1970), however, showed that high humidity was critical for successful emergence from the egg. Holling (1988) suggested that the drought moisture relationship was complex due to the differences between host and parasite responses to this variable. Our results highlighted that moisture may be a key variable but not in the traditionally expected sense.

In terms of temperature, Gray (2008) suggested that higher mean temperatures may be of greater benefit to the natural enemy complex than to the SBW by increasing the development rate, fecundity and search rate of multiple parasitoid species. However, Stireman et al. (2005) suggested that parasitoids may be disadvantaged by slower dispersal during range expansion. Régnière et al. (2012) explained that temperature limits the northern range of the insect when summers are too short for the insect to complete its life cycle but that the southern range limit is determined by warm falls that cause energy reserve exhaustion. Thus, although warmer spring and early summer temperatures can enhance larval development and feeding, warmer temperatures in the fall increase larval mortality.

This is consistent with the results of our study, where there was a negative relationship with the magnitude of insect disturbance and minimum temperature as lower minimum temperatures in the fall and winter would induce earlier and longer diapause, thus reducing mortality due to reserve exhaustion. Lower minimum temperatures may also be more disadvantageous to the natural enemies than to the SBW if the temperature extremes exceed the optimum temperature for over-wintering survival (Gray 2008). Spruce budworms have evolved several strategies to adjust to the cold weather, including extended super-cooling capacity (Han and Bauce 1993). Han and Bauce (1995) found that larvae exposed to −23°C outside had a much higher survival than the larvae maintained at +2°C. This could explain the negative relationship between DD0 and forest insect disturbance, and high tree mortality.

### Relating insect disturbance relationship with climate to tree mortality by other variables

#### Annual tree mortality and forest insect disturbance trend by province

We found that tree mortality and forest insect disturbance increased significantly from Ontario to Quebec (Table 3). In our study, tree mortality was positively related to the severity of forest insect disturbance. In general, the productivity of forest land in Quebec is higher than Ontario. Better sites sustained higher cumulative defoliation than poor or medium sites (MacLean and MacKinnon 1997). Higher defoliation on good sites is possibly in relation to higher nutrition of the foliage. Good sites tend to have higher nutrient concentrations than poor sites, potentially making them better for budworm fecundity and survival (Schmitt et al. 1983; Mattson et al. 1991), which then feeds back to higher tree mortality.

**Table 3:** Annual tree mortality and forest insect disturbance for different provinces (0: Ontario; 1: Quebec), different dominant tree species (4: balsam fir; 3: black spruce; 2: trembling aspen; 1: jack pine; 0: others), different proportions of hardwoods, different maturity classes (0: <80 years; 1: ≥80), diameter classes (0: <10 cm; 1: 10 to 15; 2: ≥15), latitude, and elevation

	Tree mortality		Forest insect disturbance		
			
Variables	*β*	*p*	*β*	*P*	*N*
Province	0.6729	<0.0001	16.5221	<0.0001	226
Dominant tree species	0.1926	<0.0001	2.0980	0.0307	226
Proportion of hardwoods	−0.8508	0.0011	−5.4959	0.0020	226
Mature or not	−0.2162	0.1026	0.0193	0.9586	226
Diameter class	0.0064	0.9571	0.0277	0.6961	226
Latitude	−0.1114	0.0210	−0.0522	0.6968	226
Elevation	0.0004	0.9200	0.0014	0.1190	226

#### Relationship between annual tree mortality and forest insect disturbance trend by dominant tree species

**Figure 4:**
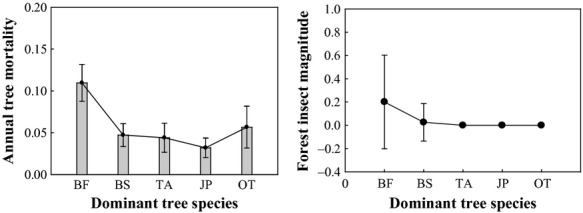
Mean annual tree mortality (mean ± 95% Conf) and forest insect disturbance (mean ± 1 SE) for different tree species (BF, Balsam fir; BS, Black spruce; TA, Trembling aspen; JP, Jack pine; OT, Others).

Annual tree mortality and forest insect magnitude increased at the 0.05 level (Table 3). We found that tree mortality of balsam fir was significantly higher than other species (*P* < 0.0001), and that balsam fir forests were the most severely disturbed (Fig. 4). Balsam fir, followed by white spruce, red spruce and black spruce are the most vulnerable trees to SBW defoliation (Blais 1983; Morin et al. 2007; Hennigar et al. 2008). Emergence of second-instar budworm larvae trends to be synchronized with bud burst of balsam fir. Balsam fir offers maximum water and nitrogen contents, as well as leaf softness at the time of bud burst (Mattson et al. 1983). Black spruce is generally considered less vulnerable to the SBW than other hosts because the late budbreak phenology of the species leads to higher larval mortality (Blais 1957; Greenbank 1963; Nealis and Régnière 2004).

#### Annual tree mortality and forest insect disturbance by stand age

The major tree species in Canada's boreal forest attain maturity at more than 80 years (Boudreault et al. 2002; Harper and Macdonald 2002). In this study, tree mortality and forest insect disturbance were not significant at the 0.05 level with the different age groups (Table 3), which indicated that forest age was not an important factor explaining the magnitude of forest insect disturbance, although some literature (e.g., Blais 1961; Mott 1963; Maclean 1980) suggests that mature stands are more susceptible to the SBW.

#### Annual tree mortality and the magnitude of forest insect disturbance by diameter classes

**Figure 5:**
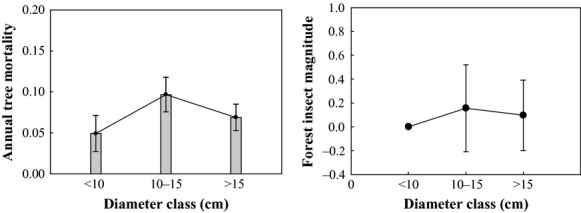
Mean annual tree mortality (mean±95% Conf) and forest insect disturbance (mean ± 1 SE) for three diameter classes.

Annual tree mortality and the magnitude of forest insect disturbance did not change with diameter class (Table 3). Medium diameter class trees had higher annual mortality rates than smaller class (*P* = 0.0022), and larger class (*P* = 0.0392). The magnitude of forest insect disturbance was less in small DBH class compared with medium DBH class stands (*P* = 0.0073) (Fig. 5). Some earlier studies have shown that tree mortality caused by SBW increases in larger diameter classes (Bergeron et al. 1995; Jardon and Doyon 2003), because small vigorous balsam fir are capable of producing greater chemical resistance against SBW attack (Bauce et al. 1994). However, higher mortality in small classes has also been observed in other studies (MacLean and Ostaff 1989; MacLean and Piene 1995). The probability of mortality of smaller DBH trees may be linked to their vigor. Our study across a wide geographic range suggests that smaller DBH trees are generally vigorous, and have higher survival than larger diameter trees.

#### Annual tree mortality and forest insect magnitude trend by latitude and elevation

Tree mortality decreased as latitude increased, but no relationship was found for the magnitude of forest insect disturbance (Table 3). The lack of a relationship with latitude was not expected, as although SBW can be found as far north as 68 degrees latitude, it rarely attains outbreak status in the northern part of its range beyond 52 degrees latitude (Rauchfuss and Svatek 2011). However, the lack of a relationship may in part be due to the SBW having stronger outbreaks in the meridional part of its range with weaker impacts to both the south and the north (Pureswaran et al., In Review[Bibr b59]). Neither mortality nor insect disturbance was affected by elevation (Table 3). Climate change may, however, modify these relationships, as Candau and Fleming (2011) and Régnière et al. (2012) have shown that with warming SBW outbreaks should shift northwards.

#### Annual tree mortality and forest insect disturbance as a function of hardwood proportion

In Canadian boreal forest, hardwoods include mainly balsam poplar, trembling aspen, and white birch, whereas softwoods include jack pine, black spruce, white spruce, larch, and balsam fir. Tree mortality and forest insect disturbance both decreased with the ratio of hardwoods (Table 3). A greater hardwood content in mixed hardwood–softwood forests reduces budworm food supply by reducing host-tree density and is one reason for reduced overall mortality. However, a protective effect of hardwoods has also been found such that the proportion of balsam fir mortality in the stand decreases as the proportion of hardwoods increases (Bergeron et al. 1995; Su et al. 1996; MacKinnon and MacLean 2003). This protective effect of hardwoods may be due to reduced oviposition by female moths and greater losses of dispersing first- and second-instar spruce budworm larvae (Kemp and Simmons 1979; Su et al. 1996) or due to a greater presence of natural enemies of the SBW (Cappuccino et al. 1998) in stands with higher hardwood content. However, whatever the mechanism, our results from across a large geographic region are consistent with local studies showing reduced host-tree mortality as the proportion of hardwoods increases.

## Conclusion

Forest insects, especially the spruce budworm, are a major disturbance in boreal forests, particularly in eastern Canada's boreal forest. Sustained defoliation results in an increase in tree mortality. Annual tree mortality was positively related forest insect disturbances. The difference in tree mortality rates between stands disturbed by insects and stands not disturbed by insects was 6.8% per year and led to a carbon sink loss of about 2.87t C ha^−1^ year^−1^ more than in natural forests. If insect-induced tree mortality continues to increase in the future, this will transform eastern Canadian boreal forests from a net carbon sink into a net carbon source. In addition, tree mortality following a SBW outbreak occurs mostly in balsam fir stands and is less for white spruce and black spruce. A high proportion of hardwoods is also related to lower SBW-caused mortality (Bergeron et al. 1995; Campbell et al. 2008). We also found that insect-induced tree mortality decreased with drought and increased with an increase in moisture in eastern Canada, which was inconsistent with previous studies (Adams et al. 2009; van Mantgem et al. 2009; Allen et al. 2010). This is because greater moisture is beneficial to balsam fir, the primary host of the SBW. We also show that tree mortality decreased with climate warming which is consistent with predictions that the southern limit of its outbreak range will move north (Régnière et al. 2012). Although the insect outbreak induces tree mortality, insect outbreak severity should be expected to decrease in eastern Canada with warmer temperatures in the future.

## Supporting Information

**Table S1.** Characteristics of the 226 forest plots.
