# Supplementary material for: Insect-induced tree mortality of boreal forests in eastern Canada under a changing climate
Source: Ecol Evol. 2014 May 16;4(12):2384–94. doi: 10.1002/ece3.988 (PMC4203287; doi:10.1002/ece3.988)
Supplement: Supplementary file 1 — Table S1. Characteristics of the 226 forest plots. [file ece30004-2384-sd1.doc]

Table S1. Characteristics of the 226 forest plots.

| Plot | Initial  age | Initial  density | Lat. ° | Long.° | Elevation | Calendar year of censuses | | | Species composition≥0.01 of Basal area |
| --- | --- | --- | --- | --- | --- | --- | --- | --- | --- |
|  |  |  |  |  |  | year1 | year2 | year3 |  |
| QC1 | 86 | 61 | 45.10 | -72.12 | 284.1 | 1997 | 2008 |  | BF 0.51, BS 0.15, EH 0.09, RM 0.07, SM 0.05, TA 0.04, AA 0.04, YB 0.04 |
| QC2 | 52 | 66 | 45.83 | -70.67 | 293 | 1978 | 1985 | 1997 | BS 0.59, BF 0.3, EWC 0.06, RM 0.04, WB 0.02 |
| QC3 | 51 | 80 | 46.21 | -70.32 | 420.5 | 1978 | 1985 |  | BF 0.42, EWC 0.36, RS 0.16, WS 0.03, YB 0.01, RM 0.01, WB 0.01 |
| QC4 | 62 | 58 | 47.05 | -70.15 | 420.3 | 1970 | 1980 |  | BF 0.52, YB 0.19, RS 0.18, RM 0.07, WB 0.02, STM 0.01 |
| QC5 | 46 | 61 | 48.18 | -71.02 | 312.1 | 1971 | 1978 | 1995 | BF 0.47, BS 0.25, TA 0.2, WS 0.07 |
| QC6 | 90 | 112 | 47.51 | -71.08 | 887.4 | 1972 | 1978 | 2003 | BF 0.91, WS 0.06, WB 0.03 |
| QC7 | 60 | 80 | 47.89 | -70.60 | 759.3 | 1978 | 1992 |  | BF 0.86, WB 0.07, WS 0.03, BS 0.03 |
| QC8 | 53 | 77 | 47.66 | -70.68 | 656.8 | 1978 | 1992 |  | BF 0.69, WB 0.17, WS 0.11, BS 0.03 |
| QC9 | 55 | 85 | 47.35 | -71.38 | 757.4 | 1978 | 1992 |  | BF 0.9, WB 0.07, WS 0.03 |
| QC10 | 43 | 84 | 47.35 | -71.38 | 757.4 | 1971 | 1978 | 1992 | BF 0.87, WS 0.08, WB 0.06 |
| QC11 | 73 | 57 | 47.45 | -71.49 | 789 | 1971 | 1978 | 1992 | BF 0.91, WB 0.07, BS 0.02 |
| QC12 | 58 | 116 | 48.00 | -71.35 | 746.5 | 1971 | 1978 | 1995 | BF 0.73, WB 0.22, WS 0.05 |
| QC13 | 51 | 61 | 48.08 | -71.57 | 505.3 | 1971 | 1978 |  | BF 0.90, BS 0.04, WB 0.02, PC 0.02, WS 0.02 |
| QC14 | 52 | 81 | 48.08 | -71.57 | 505.3 | 1971 | 1978 |  | BF 0.95, WB 0.04, BS 0.01 |
| QC15 | 75 | 63 | 48.60 | -72.58 | 300 | 1979 | 1995 |  | BF 0.5, WB 0.22, WS 0.11, TA 0.08, BS 0.07, WI 0.01 |
| QC16 | 27 | 67 | 47.12 | -70.80 | 318.5 | 1978 | 1992 |  | BF 0.96, PC 0.02, WB 0.02 |
| QC17 | 31 | 82 | 47.18 | -70.91 | 702.8 | 1978 | 1992 | 2003 | BF 0.77, AMA 0.15, WB 0.04, PC 0.02, WS 0.02 |
| QC18 | 87 | 68 | 47.27 | -70.65 | 221.7 | 1978 | 1992 |  | BF 0.82, WB 0.09, PC 0.09, WS 0.01 |
| QC19 | 54 | 59 | 47.28 | -70.83 | 925.5 | 1978 | 1992 |  | BF 0.89, WS 0.04, WB 0.03, AMA 0.03 |
| QC20 | 67 | 63 | 47.35 | -70.54 | 286.9 | 1992 | 2003 |  | TA 0.48, EWC 0.2, WB 0.15, BP 0.09, RM 0.03, SM 0.03, BF 0.01, STM 0.01 |
| QC21 | 91 | 61 | 47.50 | -70.78 | 744.1 | 1992 | 2003 |  | BF 0.66, BS 0.25, WB 0.09 |
| QC22 | 62 | 106 | 48.02 | -70.29 | 538.5 | 1971 | 1978 | 1992 | BF 0.9, BS 0.05, WB 0.05 |
| QC23 | 88 | 56 | 48.02 | -70.29 | 538.5 | 1978 | 1992 |  | BF 0.55, BS 0.41, WB 0.04 |
| QC24 | 104 | 65 | 48.05 | -70.22 | 366.6 | 1978 | 1992 |  | BF 0.51, BS 0.18, WB 0.17, WS 0.14 |
| QC25 | 116 | 58 | 50.73 | -60.72 | 249 | 1989 | 2002 |  | BS 0.64, BF 0.36 |
| QC26 | 84 | 52 | 50.73 | -60.72 | 249 | 1989 | 2002 |  | BF 0.52, BS 0.48 |
| QC27 | 109 | 68 | 50.82 | -59.96 | 177 | 1989 | 2003 |  | BF 0.61, BS 0.37, WB 0.02 |
| QC28 | 115 | 54 | 50.82 | -59.96 | 177 | 1989 | 2003 |  | BS 0.56, BF 0.36, WB 0.08 |
| QC29 | 129 | 60 | 50.83 | -59.57 | 149 | 1989 | 2003 |  | BS 0.62, BF 0.38 |
| QC30 | 74 | 72 | 50.98 | -59.51 | 125 | 1989 | 2003 |  | BF 0.86, WS 012, BS 0.02 |
| QC31 | 75 | 67 | 51.02 | -60.31 | 215 | 1989 | 2003 |  | BF 0.77, BS 0.23, WS 0.01 |
| QC32 | 109 | 75 | 51.02 | -60.31 | 215 | 1989 | 2003 |  | BF 0.87, BS 0.11, WS 0.02 |
| QC33 | 68 | 80 | 51.07 | -60.13 | 181 | 1989 | 2003 |  | BF 0.63, BS 0.14, WS 0.13, WB 0.1 |
| QC34 | 105 | 91 | 51.06 | -60.13 | 181 | 1989 | 2003 |  | BF 0.8, WS 0.12, BS 0.07, WB 0.01 |
| QC35 | 93 | 71 | 51.42 | -59.63 | 233 | 1989 | 2003 |  | BS 0.56, BF 0.41, WB 0.03 |
| QC36 | 107 | 73 | 51.12 | -61.62 | 359 | 1989 | 2002 |  | BS 0.69, BF 0.31 |
| QC37 | 94 | 52 | 46.88 | -78.83 | 281 | 1979 | 1996 |  | EH 0.43, BF 0.15, EWC 0.1, WB 0.09, YB 0.09, RM 0.07, WS 0.06, SM 0.01 |
| QC38 | 57 | 57 | 47.20 | -78.81 | 311 | 1979 | 1996 |  | TA 0.66, BS 0.25, JP 0.09 |
| QC39 | 79 | 80 | 49.73 | -77.67 | 278 | 1979 | 1996 |  | BF 0.75, BS 0.2, WS 0.05, WB 0.01 |
| QC40 | 206 | 63 | 51.08 | -59.43 | 104 | 1989 | 2003 |  | BF 0.58, BS 0.36, WB 0.06 |
| QC41 | 92 | 54 | 51.08 | -59.43 | 104 | 1989 | 2003 |  | BS 0.52, BF 0.48 |
| QC42 | 78 | 55 | 51.17 | -59.41 | 175 | 1989 | 2003 |  | BS 0.57, BF 0.43 |
| QC43 | 66 | 52 | 47.02 | -78.38 | 311 | 1979 | 1996 |  | WB 0.31, EWC 0.3, WS 0.24, BF 0.15 |
| QC44 | 91 | 71 | 47.33 | -78.56 | 334 | 1972 | 1979 | 1996 | BF 0.57, BS 0.15, WB 0.12, TA 0.1, YB 0.03, EWC 0.03 |
| QC45 | 91 | 55 | 46.88 | -79.05 | 294 | 1979 | 1996 |  | EWC 0.3, WB 0.21, BS 0.17, BF 0.14, WS 0.14, YB 0.04 |
| QC46 | 52 | 77 | 46.88 | -79.07 | 294 | 1972 | 1979 |  | PB 0.42, BF 0.31, RP 0.15, WS 0.08, BS 0.05 |
| QC47 | 51 | 62 | 46.88 | -79.13 | 306 | 1972 | 1979 | 1996 | BF 0.57, WB 0.21, TA 0.12, BS 0.09 |
| QC48 | 50 | 69 | 46.98 | -77.98 | 339 | 1972 | 1979 |  | BF 0.45, WS 0.25, WB 0.13,BS 0.1, EWC 0.05, RM 0.01 |
| QC49 | 50 | 60 | 47.17 | -78.48 | 390 | 1979 | 1996 |  | BF 0.3, BS 0.24, TA 0.18, EWC 0.15, RM 0.08, WB 0.05 |
| QC50 | 50 | 72 | 47.20 | -78.27 | 376 | 1972 | 1979 |  | BF 0.67, WB 0.15, WS 0.14, EWC 0.03, WP 0.02 |
| QC51 | 50 | 54 | 47.07 | -76.83 | 424 | 1979 | 1993 |  | BS 0.93, TA 0.07 |
| QC52 | 53 | 62 | 47.15 | -76.62 | 366 | 1972 | 1979 |  | BF 0.54, WS 0.28, TA 0.1, WS 0.07, WI 0.01, WB 0.01 |
| QC53 | 68 | 53 | 47.26 | -75.85 | 367 | 1972 | 1979 |  | BF 0.42, BS 0.22, WB 0.21, RM 0.1, WS 0.05 |
| QC54 | 88 | 67 | 47.29 | -76.84 | 377 | 1972 | 1980 |  | BF 0.47, WS 0.24, TA 0.21, WB 0.08 |
| QC55 | 81 | 52 | 47.33 | -76.46 | 313 | 1972 | 1979 |  | BF 0.48, TA 0.37, WS 0.07, WB 0.06, RM 0.02 |
| QC56 | 48 | 58 | 47.35 | -76.02 | 439 | 1979 | 1993 |  | WB 0.37, BF 0.36, EWC 0.11, RM 0.1, YB 0.03, WS 0.03 |
| QC57 | 47 | 57 | 46.72 | -77.18 | 299 | 1972 | 1979 |  | BF 0.36, EWC 0.11, RM 0.1, YB 0.03, WS 0.03 |
| QC58 | 36 | 66 | 46.64 | -77.30 | 301 | 1972 | 1979 |  | TA 0.44, BF 0.3, YB 0.12, BTA 0.09, RM 0.05, SM 0.01 |
| QC59 | 52 | 66 | 47.08 | -76.88 | 426 | 1972 | 1979 |  | BS 0.4, BF 0.31, RM 0.15, WS 0.11, WB 0.04 |
| QC60 | 64 | 67 | 47.08 | -76.89 | 426 | 1972 | 1979 |  | BF 0.57, RM 0.37, WS 0.04, WB 0.01 |
| QC61 | 67 | 55 | 47.14 | -76.97 | 365 | 1972 | 1979 |  | BF 0.5, WB 0.23, WS 0.2, RM 0.06 |
| QC62 | 51 | 60 | 47.28 | -77.42 | 364 | 1972 | 1980 |  | BF 0.29, EWC 0.28, WB 0.2, RM 0.15, YB 0.06, WS 0.02 |
| QC63 | 65 | 57 | 46.70 | -77.87 | 367 | 1972 | 1979 |  | WB 0.57, BF 0.39, WS 0.04, WS 0.01 |
| QC64 | 49 | 59 | 46.72 | -77.88 | 367 | 1972 | 1979 |  | YB 0.32, BF 0.28, BS 0.18, RM 0.14, WP 0.04, EWC 0.03, WS 0.02 |
| QC65 | 64 | 51 | 46.75 | -77.84 | 311 | 1972 | 1979 |  | WB 0.28, TA 0.27, BF 0.27, WS 0.1, WP 0.08 |
| QC66 | 73 | 54 | 46.75 | -77.85 | 311 | 1972 | 1979 |  | EWC 0.39, BF 0.25, WB 0.15, BS 0.12, WS 0.08 |
| QC67 | 54 | 70 | 46.67 | -77.53 | 364 | 1972 | 1979 |  | BF 0.64, BS 0.35, EWC 0.01 |
| QC68 | 57 | 59 | 46.72 | -77.60 | 423 | 1972 | 1979 |  | BF 0.72, BS 0.15, WS 0.09, WB 0.03 |
| QC69 | 87 | 52 | 46.90 | -77.85 | 373 | 1972 | 1979 |  | TA 0.23, WS 0.23, WB 0.22, BF 0.22, WS 0.06, RM 0.04 |
| QC70 | 91 | 56 | 46.90 | -77.85 | 373 | 1972 | 1979 |  | WB 0.35, BF 0.33, WS 0.19, BS 0.07, TA 0.06 |
| QC71 | 67 | 59 | 47.00 | -77.78 | 377 | 1972 | 1979 | 1993 | BF 0.46, TA 0.39, WB 0.08, WS 0.04, EWC 0.03, BS 0.01 |
| QC72 | 47 | 54 | 47.05 | -77.72 | 365 | 1972 | 1979 |  | BF 0.7, WS 0.12, BS 0.11, WB 0.07 |
| QC73 | 50 | 57 | 47.03124 | -77.75 | 365 | 1979 | 1993 |  | BF 0.24, EWC 0.23, WB 0.22, TA 0.17, RM 0.08, WS 0.06 |
| QC74 | 56 | 84 | 47.11 | -77.87 | 365 | 1972 | 1979 |  | BS 0.74, BF 0.15, EWC 0.07, WB 0.04 |
| QC75 | 54 | 83 | 47.30 | -77.93 | 363 | 1972 | 1980 |  | BF 0.81, WB 0.14, BS 0.05 |
| QC76 | 47 | 69 | 47.39 | -78.06 | 361 | 1972 | 1981 |  | BF 0.87, WB 0.12, AMA 0.01 |
| QC77 | 41 | 59 | 47.17 | -74.47 | 442 | 1980 | 1997 |  | BF 0.58, PC 0.31, WB 0.11 |
| QC78 | 72 | 66 | 47.83 | -75.79 | 432 | 1973 | 1980 | 1993 | BF 0.68, WB 0.18, WS 0.11, TA 0.04 |
| QC79 | 69 | 54 | 47.85 | -74.60 | 418 | 1973 | 1980 |  | BF 0.31, WS 0.19, TA 0.17, WB 0.14, JP 0.13, BS 0.04, RM 0.03 |
| QC80 | 39 | 83 | 47.25 | -74.95 | 382 | 1980 | 1997 | 2006 | WB 0.44, BF 0.34, PC 0.1, YB 0.06, WS 0.04, RM 0.03 |
| QC81 | 36 | 60 | 48.09 | -71.89 | 486.8 | 1980 | 1995 |  | BS 0.79, BF 0.16, WB 0.04, TA 0.01 |
| QC82 | 34 | 62 | 48.19 | -73.95 | 569 | 1974 | 1980 |  | BF 0.81, WB 0.16, RS 0.02 |
| QC83 | 65 | 51 | 48.51 | -73.70 | 588 | 1975 | 1980 |  | BF 0.85, WS 0.06, WB 0.06, WS 0.03 |
| QC84 | 45 | 65 | 47.38 | -69.71 | 410 | 1981 | 1985 | 1992 | BF 0.75, TA 0.22, BS 0.03 |
| QC85 | 72 | 53 | 48.11 | -67.21 | 251 | 1995 | 2003 |  | BF 0.53, WB 0.22, YB 0.18, RM 0.05, WS 0.02 |
| QC86 | 89 | 55 | 48.72 | -66.98 | 322 | 1975 | 1986 |  | BF 0.6, EWC 0.27, BS 0.08, WB 0.02, PC 0.02, YB 0.01 |
| QC87 | 90 | 52 | 48.03 | -79.12 | 277 | 1975 | 1981 |  | BF 0.51, EWC 0.39, WS 0.1, WB 0.01 |
| QC88 | 49 | 62 | 48.23 | -79.33 | 288 | 1975 | 1981 |  | BF 0.78, WB 0.22 |
| QC89 | 77 | 58 | 48.48 | -79.16 | 277 | 1975 | 1981 |  | BF 0.49, WS 0.25, WB 0.18, BS 0.09 |
| QC90 | 73 | 56 | 48.50 | -79.42 | 278 | 1981 | 1996 |  | BF 0.67, EWC 0.21, WB 0.12 |
| QC91 | 81 | 66 | 48.50 | -79.42 | 278 | 1981 | 1996 |  | BF 0.5, WS 0.29, WB 0.18, BS 0.04 |
| QC92 | 51 | 69 | 48.25 | -66.52 | 412 | 1975 | 1986 | 1996 | BF 0.77, WS 0.14, YB 0.08, WB 0.01 |
| QC93 | 61 | 97 | 48.30 | -66.65 | 430 | 1975 | 1986 |  | BF 0.85, WB 0.07, WS 0.08 |
| QC94 | 92 | 81 | 48.33 | -66.57 | 154 | 1975 | 1986 |  | BF 0.92, WS 0.06, WB 0.02 |
| QC95 | 48 | 53 | 47.45 | -77.18 | 365 | 1975 | 1980 |  | BF 0.59, PIR 0.18, WB 0.08, TA 0.08, BS 0.06 |
| QC96 | 92 | 58 | 47.98 | -76.00 | 482 | 1980 | 1993 |  | WB 0.45, BF 0.44, WS 0.07, BS 0.04 |
| QC97 | 91 | 59 | 48.43 | -66.32 | 420 | 1976 | 1986 |  | BF 0.77, WB 0.11, BS 0.07, WS 0.06 |
| QC98 | 80 | 65 | 48.80 | -66.35 | 558 | 1986 | 1996 |  | BF 0.8, WS 0.17, BS 0.03 |
| QC99 | 59 | 136 | 48.80 | -66.34 | 555 | 1986 | 1996 |  | BF 0.94, WS 0.06 |
| QC100 | 60 | 86 | 48.67 | -64.39 | 162 | 1976 | 1986 |  | BS 0.5, BF 0.42, WB 0.04, WS 0.03 |
| QC101 | 90 | 77 | 48.88 | -66.73 | 175 | 1976 | 1986 |  | BF 0.76, EWC 0.2, AMA 0.04 |
| QC102 | 92 | 88 | 48.90 | -65.92 | 429 | 1976 | 1986 |  | BF 1.00 |
| QC103 | 61 | 100 | 48.97 | -65.87 | 429 | 1976 | 1986 |  | BF 0.79, WS 0.21 |
| QC104 | 78 | 51 | 49.20 | -65.55 | 505 | 1976 | 1986 |  | BF 0.54, BS 0.3, EWC 0.15 |
| QC105 | 82 | 60 | 48.18 | -69.72 | 151 | 1976 | 1985 |  | WS 0.27, WB 0.24, BF 0.21, BS 0.12, TA 0.1, RM 0.05, STM 0.01 |
| QC106 | 71 | 54 | 48.43 | -70.26 | 407.5 | 1976 | 1985 |  | BF 0.83, WS 0.15, WB 0.02 |
| QC107 | 62 | 77 | 48.45 | -70.25 | 407.5 | 1976 | 1985 |  | BF 0.71, WS 0.24, WB 0.06 |
| QC108 | 89 | 53 | 48.45 | -70.30 | 407.5 | 1976 | 1985 |  | BF 0.79, BS 0.12, WS 0.05, WB 0.03 |
| QC109 | 89 | 64 | 48.45 | -70.30 | 407.5 | 1976 | 1985 |  | BF 0.85, WS 0.08, BS 0.07, WB 0.01 |
| QC110 | 91 | 54 | 48.47 | -70.58 | 395 | 1976 | 1987 |  | BF 0.82, BS 0.12, WB 0.04, RM 0.03 |
| QC111 | 46 | 51 | 46.47 | -73.78 | 489 | 1976 | 1987 |  | BF 0.62, TA 0.19, WB 0.14, BS 0.05 |
| QC112 | 57 | 58 | 46.07 | -74.03 | 358 | 1997 | 2008 |  | BTA 0.5, BF 0.4, TA 0.08, RS 0.02, RM 0.01 |
| QC113 | 84 | 59 | 48.58 | -69.95 | 487.7 | 1976 | 1985 |  | BF 0.92, WS 0.05, BS 0.02 |
| QC114 | 76 | 52 | 48.59 | -69.95 | 644.5 | 1976 | 1985 |  | BF 0.72, WS 0.2, WB 0.08 |
| QC115 | 58 | 68 | 48.72 | -69.85 | 509.1 | 1976 | 1985 |  | BF 0.69, BS 0.25, WS 0.06 |
| QC116 | 76 | 56 | 48.74 | -70.06 | 797.1 | 1976 | 1985 |  | BF 0.82, WS 0.07, BS 0.06, WB 0.04 |
| QC117 | 96 | 53 | 48.75 | -69.45 | 337.1 | 1976 | 1985 |  | BS 0.7, BF 0.25, WB 0.05 |
| QC118 | 51 | 117 | 48.75 | -70.02 | 797.1 | 1975 | 1985 |  | BF 0.81, WS 0.14, BS 0.03, WB 0.02 |
| QC119 | 58 | 81 | 49.01 | -70.87 | 639 | 1976 | 1987 |  | BF 0.99, BS 0.01 |
| QC120 | 69 | 57 | 49.17 | -70.88 | 551 | 1976 | 1987 |  | BF 0.84, BS 0.14, WB 0.02 |
| QC121 | 109 | 65 | 46.67 | -73.28 | 303 | 1986 | 1997 |  | BF 0.56, WB 0.15, YB 0.14, RS 0.1, EWC 0.05 |
| QC122 | 57 | 83 | 46.57 | -73.71 | 545 | 1987 | 1997 |  | BS 0.79, TA 0.17, BF 0.03, PC 0.01 |
| QC123 | 70 | 59 | 46.53 | -74.46 | 453 | 1976 | 1984 |  | BS 0.91, BF 0.09 |
| QC124 | 62 | 88 | 47.17 | -71.92 | 614 | 1976 | 1986 |  | BF 0.98, WS 0.02 |
| QC125 | 69 | 100 | 47.17 | -71.91 | 614 | 1976 | 1986 |  | BF 0.92, WB 0.06, BS 0.02 |
| QC126 | 30 | 55 | 47.20 | -71.83 | 614 | 1976 | 1986 |  | BF 0.96, AMA 0.03, WS 0.01 |
| QC127 | 96 | 66 | 47.28 | -71.58 | 709.5 | 1976 | 1986 |  | BF 0.79, WS 0.14, WB 0.07 |
| QC128 | 55 | 54 | 47.25 | -72.03 | 476 | 1976 | 1986 |  | BF 0.72, YB 0.24, WB 0.03, PC 0.01 |
| QC129 | 72 | 62 | 47.38 | -72.18 | 461 | 1987 | 1997 |  | BS 0.76, BF 0.22 |
| QC130 | 38 | 51 | 47.65 | -73.15 | 309 | 1976 | 1987 |  | BF 0.68, YB 0.24, WB 0.05, TA 0.01, RM 0.01 |
| QC131 | 50 | 55 | 47.65 | -73.34 | 280 | 1976 | 1987 |  | WB 0.35, BF 0.34, TA 0.25, BS 0.06 |
| QC132 | 81 | 69 | 48.48 | -64.93 | 413 | 1997 | 2005 |  | BF 0.67, WS 0.31, BS 0.01, WB 0.01 |
| QC133 | 40 | 67 | 48.55 | -64.90 | 391 | 1976 | 1986 | 1996 | BF 0.82, BS 0.1, WB 0.07, WS 0.01 |
| QC134 | 87 | 70 | 48.58 | -66.28 | 660 | 1976 | 1986 |  | BS 0.53, BF 0.47 |
| QC135 | 60 | 61 | 49.48 | -67.48 | 79 | 1977 | 1989 |  | BF 0.81, WB 0.16, WS 0.04 |
| QC136 | 65 | 67 | 49.49 | -67.49 | 79 | 1977 | 1989 |  | BF 0.83, WS 0.09, WB 0.08 |
| QC137 | 98 | 57 | 49.68 | -67.60 | 449 | 1977 | 1989 |  | BF 0.52, WB 0.46, BS 0.02 |
| QC138 | 101 | 54 | 50.30 | -64.75 | 54 | 1989 | 2002 |  | BF 0.42, BS 0.41, WB 0.07, MEL 0.06, WS 0.05 |
| QC139 | 136 | 64 | 50.32 | -63.85 | 45 | 1989 | 2002 |  | BF 0.69, WS 0.23, BS 0.07 |
| QC140 | 90 | 54 | 47.00 | -69.89 | 366 | 1993 | 2003 |  | EWC 0.95, WB 0.04, BF 0.01 |
| QC141 | 50 | 69 | 48.21 | -67.84 | 452 | 1995 | 2003 |  | BF 0.62, BS 0.23, WS 0.15 |
| QC142 | 103 | 64 | 48.77 | -70.42 | 784.8 | 1998 | 2007 |  | BF 0.82, WS 0.08, BS 0.08, WB 0.01 |
| QC143 | 73 | 53 | 48.79 | -70.45 | 686 | 1988 | 1998 |  | BF 0.65, WB 0.32, WS 0.03 |
| QC144 | 82 | 72 | 48.85 | -65.79 | 537 | 1988 | 1997 |  | BF 0.65, WS 0.26, WB 0.09 |
| QC145 | 58 | 57 | 49.01 | -65.86 | 490 | 1996 | 2003 |  | BF 0.62, WS 0.34, AMA 0.03, WB 0.01 |
| QC146 | 76 | 66 | 49.38 | -67.82 | 203 | 1989 | 1999 |  | BF 051, BS 0.47, AMA 0.02 |
| QC147 | 96 | 84 | 49.47 | -67.75 | 222 | 1989 | 1999 |  | BS 0.53, BF 0.45, AMA 0.02 |
| QC148 | 99 | 54 | 49.47 | -67.74 | 222 | 1989 | 1999 |  | BF 0.49, BS 0.46, WB 0.06 |
| QC149 | 69 | 52 | 49.62 | -67.87 | 354 | 1989 | 1999 |  | BF 0.43, WB 0.29, WS 0.16, BS 0.13 |
| QC150 | 154 | 77 | 50.40 | -62.40 | 65 | 1989 | 2002 |  | BS 0.58, BF 0.42 |
| QC151 | 99 | 59 | 50.57 | -64.40 | 135 | 1989 | 2002 |  | BF 0.71, BS 0.22, WB 0.05, WS 0.02 |
| QC152 | 99 | 74 | 50.58 | -62.74 | 98 | 1989 | 2002 |  | BF 0.47, BS 0.42, WB 0.07, WS 0.04 |
| QC153 | 79 | 52 | 50.60 | -62.80 | 160 | 1989 | 2002 |  | BF 0.62, BS 0.35, WB 0.03 |
| QC154 | 167 | 69 | 50.65 | -62.63 | 176 | 1989 | 2002 |  | BF 0.82, BS 0.1, WB 0.08 |
| QC155 | 96 | 88 | 50.62 | -59.58 | 61 | 1989 | 2003 |  | BF 0.98, WS 0.02 |
| QC156 | 99 | 61 | 51.45 | -58.68 | 53 | 1989 | 2003 |  | BF 0.69, BS 0.31 |
| QC157 | 92 | 59 | 51.57 | -58.95 | 226 | 1989 | 2003 |  | BF 0.99, BS 0.01 |
| QC158 | 45 | 56 | 48.47 | -66.57 | 562 | 1989 | 1996 |  | BF 0.81, WS 0.15, WB 0.04 |
| QC159 | 55 | 52 | 48.92 | -69.16 | 145.7 | 1989 | 1999 |  | BF 0.86, YB 0.08, WB 0.05, WS 0.01 |
| QC160 | 74 | 71 | 49.84 | -68.20 | 349 | 1989 | 2001 |  | BF 0.87, WB 0.09, BS 0.04 |
| QC161 | 43 | 75 | 48.15 | -66.82 | 291 | 1990 | 1997 | 2003 | BF 0.65, WB 0.18, WS 0.12, PC 0.05 |
| QC162 | 147 | 56 | 50.28 | -65.03 | 138 | 1990 | 2002 |  | BS 0.52, BF 0.48 |
| QC163 | 82 | 57 | 48.06 | -66.55 | 233 | 1991 | 1997 |  | BF 0.29, WB 0.18, EWC 0.15, WS 0.09, BP 0.08, WI 0.08, TA 0.07, RM 0.06 |
| QC164 | 63 | 55 | 48.12 | -71.02 | 427.6 | 1991 | 2007 |  | JP 0.71, BS 0.27, BF 0.02 |
| QC165 | 92 | 51 | 48.66 | -66.09 | 242 | 1991 | 1997 | 2003 | BS 0.6, BF 0.4 |
| QC166 | 82 | 56 | 47.68 | -71.12 | 914 | 1992 | 2003 |  | BF 0.58, BS 0.31, WB 0.11 |
| QC167 | 55 | 52 | 47.19 | -71.46 | 673.3 | 1992 | 2003 |  | BF 0.58, WB 0.29, AMA 0.11, PC 0.02 |
| QC168 | 86 | 62 | 49.10 | -77.00 | 272 | 1992 | 2007 |  | BS 0.83, JP 0.1, TA 0.07 |
| QC169 | 60 | 68 | 47.27 | -71.18 | 610.8 | 2000 | 2005 |  | BF 0.82, WB 0.13, WS 0.05 |
| QC170 | 61 | 83 | 48.76 | -64.17 | 47 | 1995 | 2006 |  | WS 0.54, BF 0.39, WB 0.05, RS 0.02 |
| QC171 | 99 | 73 | 48.80 | -64.24 | 47 | 1995 | 2006 |  | BF 0.52, EWC 0.17, WB 0.15, RM 0.09, WS 0.07, STM 0.01 |
| QC172 | 97 | 62 | 49.21 | -65.78 | 11 | 1997 | 2003 |  | BF 0.46, WB 0.33, WS 0.15, AMA 0.06 |
| QC173 | 63 | 61 | 47.33 | -71.10 | 610.8 | 2001 | 2007 |  | BF 0.92, BS 0.05, WB 0.03 |
| QC174 | 60 | 63 | 47.27 | -71.17 | 610.8 | 2003 | 2008 |  | BF 0.88, WS 0.1, WB 0.02, BS 0.01 |
| QC175 | 58 | 67 | 47.27 | -71.15 | 610.8 | 2003 | 2008 |  | BF 0.83, WB 0.12, AMA 0.05 |
| QC176 | 23 | 62 | 47.23 | -71.17 | 701.2 | 2003 | 2008 |  | BF 0.46, WS 0.35, WB 0.17, BS 0.03 |
| ON1 | 86 | 138 | 50.72 | -93.94 | 362 | 1993 | 1998 |  | TA 0.71, JP 0.12, WS 0.08, BF 0.05, BS 0.04 |
| ON2 | 70 | 226 | 51.17 | -93.83 | 361 | 1993 | 1998 |  | JP 0.99, BS 0.01 |
| ON3 | 120 | 545 | 50.94 | -93.19 | 413 | 1993 | 1998 |  | TA 0.56, BS 0.23, BF 0.21 |
| ON4 | 17 | 695 | 50.65 | -92.97 | 387 | 1993 | 1998 |  | JP 0.95, TA 0.04, BF 0.01 |
| ON5 | 40 | 493 | 50.66 | -93.05 | 387 | 1993 | 1998 |  | BF 0.37, WB 0.29, TA 0.13, JP 0.13, BS 0.07 |
| ON6 | 96 | 231 | 47.66 | -81.81 | 370 | 1994 | 1999 |  | BS 0.89, WB 0.06, BF 0.05 |
| ON7 | 162 | 221 | 49.73 | -83.90 | 242 | 1992 | 1997 |  | BS 1.00 |
| ON8 | 109 | 497 | 49.65 | -84.57 | 342 | 1992 | 1997 |  | BS 1.00 |
| ON9 | 22 | 614 | 49.61 | -84.56 | 342 | 1993 | 1997 |  | TA 0.56, JP 0.4, WB 0.03, BF 0.01 |
| ON10 | 111 | 100 | 49.42 | -84.71 | 305 | 1993 | 1997 |  | JP 0.7, BS 0.22, BF 0.05, WS 0.03 |
| ON11 | 72 | 98 | 48.29 | -79.87 | 347 | 1993 | 1999 |  | JP 0.99, BS 0.01 |
| ON12 | 70 | 321 | 48.00 | -80.33 | 309 | 1994 | 1999 |  | JP 0.42, BS 0.42, TA 0.15, BF 0.01 |
| ON13 | 86 | 166 | 47.71 | -80.41 | 307 | 1994 | 1999 |  | JP 0.55, BS 0.31, TA 0.08, BF 0.05, WB 0.01 |
| ON14 | 79 | 258 | 47.70 | -80.34 | 307 | 1994 | 1999 |  | BS 0.54, JP 0.38, TA 0.04, WB 0.03, BF 0.01 |
| ON15 | 85 | 109 | 48.53 | -80.28 | 316 | 1994 | 1999 |  | TA 0.59, BP 0.41 |
| ON16 | 74 | 54 | 48.60 | -80.37 | 276 | 1994 | 1999 |  | TA 1.00 |
| ON17 | 81 | 111 | 50.71 | -91.87 | 400 | 1993 | 1998 |  | TA 0.59, BP 0.17, BS 0.1, JP 0.08, TAM 0.05 |
| ON18 | 153 | 278 | 50.31 | -90.49 | 412 | 1993 | 1998 |  | JP 0.61, BS 0.35, TA 0.02, WB 0.02 |
| ON19 | 115 | 86 | 49.45 | -91.49 | 415 | 1993 | 1998 |  | TA 0.54, wb 0.26, RM 0.09, BS 0.05, JP 0.03, WS 0.02, BF 0.01 |
| ON20 | 68 | 147 | 50.26 | -91.22 | 400 | 1993 | 1998 |  | TA 0.46, JP 0.29, BS 0.23 WS 0.02, BF 0.01 |
| ON21 | 56 | 203 | 49.33 | -88.04 | 442 | 1994 | 1999 |  | BF 0.39, WB 0.30, WS 0.27, BS 0.04 |
| ON22 | 58 | 189 | 49.34 | -88.04 | 442 | 1994 | 1999 |  | BF 0.44, WS 0.27, WB 0.25, BS 0.05 |
| ON23 | 74 | 447 | 49.51 | -87.63 | 418 | 1994 | 1999 |  | BF 0.42, TA 0.21, WS 0.13, WB 0.12, WC 0.06, BS 0.05 |
| ON24 | 78 | 248 | 49.52 | -87.63 | 418 | 1994 | 1999 |  | BS 0.5, WB 0.23, BF 0.19, WS 0.07, TA 0.01 |
| ON25 | 144 | 295 | 49.50 | -87.44 | 395 | 1994 | 1999 |  | BS 0.68, WC 0.31, BF 0.01 |
| ON26 | 105 | 230 | 49.44 | -87.75 | 419 | 1994 | 1999 |  | BS 0.76, TA 0.13, WB 0.05, BF 0.06 |
| ON27 | 165 | 162 | 49.44 | -87.75 | 419 | 1994 | 1999 |  | BS 0.9, WC 0.06, BF 0.03, WB 0.01 |
| ON28 | 96 | 179 | 48.72 | -89.37 | 454 | 1992 | 1997 |  | BS 0.79, TA 0.17, BF 0.03, BP 0.01 |
| ON29 | 11 | 114 | 48.90 | -89.02 | 468 | 1992 | 1997 |  | JP 0.88, TA 0.1, BF 0.01, BS 0.01 |
| ON30 | 140 | 188 | 49.24 | -88.18 | 276 | 1994 | 1999 |  | WP 0.46, TA 0.3, WB 0.11, BF 0.11, BS 0.01, WS 0.01 |
| ON31 | 76 | 112 | 49.03 | -88.31 | 268 | 1992 | 1997 |  | JP 0.81, TA 0.13, BF 0.02, WB 0.02, BS 0.02, WS 0.01 |
| ON32 | 77 | 69 | 48.22 | -90.08 | 419 | 1992 | 1997 |  | TA 1.00 |
| ON33 | 70 | 180 | 48.16 | -89.88 | 426 | 1992 | 1997 |  | JP 0.87, BF 0.07, WS 0.03, WB 0.01, BS 0.01 |
| ON34 | 73 | 98 | 48.16 | -89.88 | 426 | 1992 | 1997 |  | JP 0.97, BF 0.03 |
| ON35 | 49 | 238 | 48.42 | -90.15 | 477 | 1992 | 1997 |  | BS 0.89, BF 0.07, TAM 0.04 |
| ON36 | 64 | 879 | 48.19 | -89.67 | 357 | 1992 | 1997 |  | BS 0.98, TAM 0.02 |
| ON37 | 68 | 91 | 48.21 | -90.45 | 487 | 1992 | 1997 |  | TA 0.9, BS 0.06, WB 0.03, JP 0.01 |
| ON38 | 56 | 219 | 48.26 | -90.58 | 430 | 1992 | 1997 |  | JP 0.9, TA 0.06, WB 0.02, BF 0.01 |
| ON39 | 167 | 145 | 48.21 | -90.30 | 515 | 1992 | 1997 |  | BS 1.00 |
| ON40 | 72 | 143 | 48.20 | -90.30 | 515 | 1992 | 1997 |  | TA 0.92, WB 0.04, BF 0.04 |
| ON41 | 71 | 62 | 48.63 | -89.74 | 427 | 1992 | 1997 |  | TA 1.00 |
| ON42 | 22 | 340 | 47.47 | -81.63 | 395 | 1993 | 1998 |  | JP 1.00 |
| ON43 | 16 | 367 | 48.60 | -81.62 | 274 | 1993 | 1998 |  | JP 1.00 |
| ON44 | 88 | 235 | 48.16 | -81.27 | 348 | 1993 | 1998 |  | JP 0.79, BS 0.10, BF 0.05, WB 0.04, TA 0.02 |
| ON45 | 56 | 544 | 47.45 | -81.41 | 396 | 1993 | 1998 |  | BS 0.95, JP 0.04, BF 0.01 |
| ON46 | 25 | 721 | 48.47 | -80.82 | 289 | 1993 | 1998 |  | JP 0.44, TA 0.41, BF 0.06, WB 0.05, BS 0.02 |
| ON47 | 17 | 289 | 48.44 | -81.54 | 317 | 1993 | 1998 |  | TA 0.92. BP 0.07, BF 0.01 |
| ON48 | 26 | 292 | 48.41 | -81.76 | 315 | 1993 | 1998 |  | WB 0.45, TA 0.24, WS 0.17, BF 0.13 |
| ON49 | 45 | 627 | 48.14 | -81.16 | 343 | 1993 | 1998 |  | BS 0.46, BF 0.22, WB 0.17, JP 0.13, WS 0.02 |
| ON50 | 66 | 383 | 49.08 | -84.76 | 337 | 1993 | 1997 |  | JP 0.43, BS 0.36, TA 0.18, WB 0.02 |

* ON=Ontario, QC=Quebec.

ξ Species composition of the stand at the first census. Values represent the proportion of the basal areas of each species and may not add to 1 due to rounding. AA=American ash (*Fraxinus nigra americana*), AMA=American mountain-ash (*Sorbus americana*), BF=Balsam fir (*Abies balsamea*), BP=Balsam popular (*Populus balsamifera*), BS=Black spruce (*Picea mariana*), BTA=Bigtooth aspen (*Populus grandidentata*), EH=Eastern hemlock (Tsuga canadensis), EWC=Eastern white cedar (*Thuja occidentalis*), JP=Jack pine (*Pinus banksiana*), PC=Pin cherry (*Prunus pensylvanica*), RM=Red maple (*Acer rubrum*), RS=Red spruce (*Picea rubens*), SM=Sugar maple (*Acer saccharum*), STM=Striped maple (*Acer pensylvanicum*), TA=Trembling aspen (*Populus tremuloides*), TAM=Tamarack (*Larix laricina*), WI=Willow (*Salix*), WB=Paper-White Birch (*Resinifera-Papyrifera betula*), WP=White pine (*Pinus strobus*), WS=White spruce (*Picea glauca*), YB=Yellow birch (*Betula alleghaniensis*).
